# Upstaged from cT1a-c to pT2a lung cancer, related to visceral pleural invasion patients, after segmentectomy: is it an indication to complete resection to lobectomy?

**DOI:** 10.1093/icvts/ivad102

**Published:** 2023-06-09

**Authors:** Joseph Lula Lukadi, Alessio Vincenzo Mariolo, Emrah Gokay Ozgur, Dominique Gossot, Jean-Marc Baste, Bertrand De Latour, Agathe Seguin-Givelet

**Affiliations:** Thoracic Department, Curie-Montsouris Thoracic Institute, Institut Mutualiste Montsouris, Paris, France; Thoracic Department, Curie-Montsouris Thoracic Institute, Institut Mutualiste Montsouris, Paris, France; Faculty of Medicine, Department of Biostatistics, Marmara University, Istanbul, Turkey; Thoracic Department, Curie-Montsouris Thoracic Institute, Institut Mutualiste Montsouris, Paris, France; Thoracic Surgery Department, Rouen University Hospital, Rouen, France; Normandie University UNIROUEN, Rouen, France; Thoracic and Cardiovascular Surgery Department, Rennes University Hospital, Rennes, France; Thoracic Department, Curie-Montsouris Thoracic Institute, Institut Mutualiste Montsouris, Paris, France; Faculty of Medecine SMBH, Paris 13 University, Sorbonne Paris cité Bobiny, Bobigny, France

**Keywords:** Segmentectomy, Visceral pleural invasion, Early-stage lung cancer

## Abstract

**OBJECTIVES:**

Segmentectomy may be indicated for T1a-cN0 non-small-cell lung cancer. However, several patients are upstaged pT2a at final pathological examination due to visceral pleural invasion (VPI). As resection is usually not completed to lobectomy, this may raise issue of potential worse prognosis. The aim of this study is to compare prognosis of VPI upstaged cT1N0 patients operated on by segmentectomy or lobectomy.

**METHODS:**

Data of patients from 3 centres were analysed. This was a retrospective study, of patients operated on from April 2007 to December 2019. Survival and recurrence were assessed by Kaplan–Meier method and cox regression analysis.

**RESULTS:**

Lobectomy and segmentectomy were performed in 191 (75.4%) and in 62 (24.5%) patients, respectively. No difference in 5-year disease-free survival rate between lobectomy (70%) and segmentectomy (64.7%) was observed. There was no difference in loco-regional recurrence, nor in ipsilateral pleural recurrence. The distant recurrence rate was higher (*P* = 0.027) in the segmentectomy group. Five-year overall survival rate was similar for both lobectomy (73%) and segmentectomy (75.8%) groups. After propensity score matching, there was no difference in 5-year disease-free survival rate (*P* = 0.27) between lobectomy (85%) and segmentectomy (66.9%), and in 5-year overall survival rate (*P* = 0.42) between the 2 groups (lobectomy 76.3% vs segmentectomy 80.1%). Segmentectomy was not impacting neither recurrence, nor survival.

**CONCLUSIONS:**

Detection of VPI (pT2a upstage) in patients who underwent segmentectomy for cT1a-c non-small-cell lung cancer does not seem to be an indication to extend resection to lobectomy.

**Figure:**
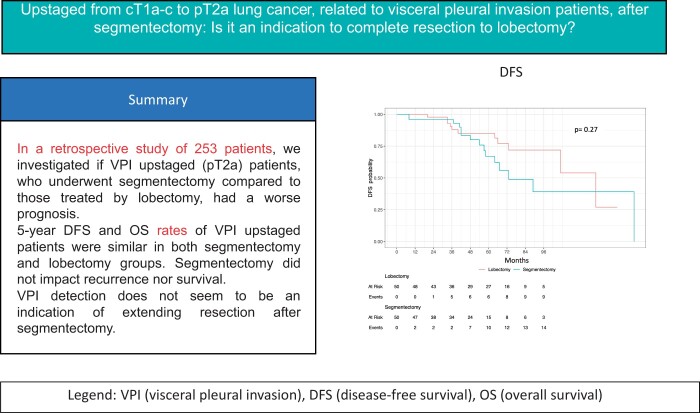

## INTRODUCTION

Early-stage non-small-cell lung cancer (NSCLC) are increasingly treated by segmentectomy [1–4]. This trend is most likely going to increase after the publication of the Japan Clinical Oncology Group 0802, a phase 3 clinical trial that showed that segmentectomy was non-inferior to lobectomy in patients with small-sized peripheral NSCLC (with consolidation-to-tumour ratio >0.5) [5]. This study and the results of the Cancer and Leukaemia group B 140503 [6] advocate segmentectomy as the surgical standard procedure for this population.

Nevertheless, visceral pleural invasion (VPI) as other pathological invasive characteristics such as lymphatic invasion, vascular invasion and spread through air spaces are correlated with a worse prognosis [7–10]. These pathological factors can be found in small-sized NSCLC, and they are only asserted at final pathological examination.

VPI is the single pathological factor that upstages cT1 to pT2a, according to the 8th Union for International Cancer Control tumour, node, metastasis (TNM) classification [11]. Its incidence in early-stage NSCLC ranges between 10 and 38% [2–4, 10, 12–15]. Some patients with small-sized NSCLC that underwent segmentectomy are found with VPI. However, cT2a and pT2a NSCLC should theoretically be treated by lobectomy [16]; this raises the issue of extending resection to lobectomy in patients who underwent a segmentectomy. We aimed at investigating in a standard practice, outside of study protocol:

- If VPI upstaged cT1N0 patients have a worse prognosis when they were treated by segmentectomy compared to patients operated on by lobectomy.
- If segmentectomy would negatively influence the long-term prognosis in VPI upstaged patients.

## MATERIALS AND METHODS

### Ethics statement

This study was approved by the ethical committee for clinical research of the French society of thoracic and cardiovascular surgery (CERC-SFCTCV-2022–06-09_21777_SEAG).

### Study cohort

We conducted a multicentric retrospective study. Data of patients operated on from April 2007 to December 2019 at Curie-Montsouris Thoracic Institute, Rouen University Hospital, and Rennes University Hospital were collected from the French national database, Epithor and from patients’ records. Patients who underwent either lobectomy or segmentectomy for early-stage NSCLC (cT1N0) with VPI at final pathological examination (pT2aN0M0, R0), were considered. All other pT2a upstaged patients’ categories, for whatever reason, were excluded.

The following data were collected: type of operation (lobectomy or segmentectomy), clinical features (gender, age, body mass index, performance status, comorbidities, smocking history, clinical stage), morphological assessments (computed tomography, positron emission tomography, brain magnetic resonance imagering, 3D reconstruction for planned segmentectomies), first second of forced expiratory volume, diffusing capacity for carbon monoxide, intraoperative characteristics (duration of surgery), post-operative traits (final pathological examination, duration of pleural drainage, length of hospital stay, complications, adjuvant chemotherapy) and follow-up parameters (recurrence and survival). Ninety postoperative days complications were assessed according to Clavien–Dindo classification, grade III or more were considered as major complications [17].

Segmentectomy was mainly preferred to lobectomy in cases of clinically less invasive pulmonary lesions or in patients with suboptimal respiratory function or presenting with major comorbidities. However, in our series, all patients who underwent segmentectomy could have supported a lobectomy.

Tumours were assessed using the 8th edition of the TNM classification. To assess pleural invasion, in pathological examination, the following stains were used: elastin at Curie-Montsouris Thoracic Institute; routinely haematoxylin eosin safran and if more challenging cases orcein at Rouen University hospital and orcein at Rennes University Hospital.

### Follow-up

Patients’ records were discussed at the oncological multidisciplinary board based on the result of final pathological examination. Patients were seen by surgeons at 30 postoperative days appointment. They were then regularly followed by their pneumologist or oncologist. They were generally checked up every 6 months for the first 2 years and then annually. The evaluation included physical examination, imaging (mostly chest-upper abdomen computed tomography scan, sometimes positron emission tomography scan or magnetic resonance imaging if needed). Recurrence was classified as follow: loco-regional recurrence (in the ipsilateral lung which included the resection margins of the lung or the bronchus, hilar lymph nodes, mediastinal lymph nodes, pleural invasion), distant recurrence (in contralateral lung parenchyma or extra thoracic metastasis).

### Statistics

Statistical analysis were performed with IBM Corp, 2016. IBM SPSS statistics for windows, version 24.0. Armonk, New York and R software version 4.2.2. Numerical variables were expressed as median and interquartile range (IQR), mean and standard deviation, categorical variables as number and percentage. We did have a couple of missing data for the following variables: duration of operation, smoking package-year and maximum standardized uptake value, they were addressed by exclusion. Disease-free survival (DFS) was defined as the time from the date of surgery until the date of recurrence, death, or last follow-up visit. Overall survival (OS) was defined as the time from the date of surgery until the date of death due to any cause or until the last follow-up visit.

Chi-squared test and Fischer’s test were used to compare categorical variables, Levene’s test analysed equality of variances, Student’s *t*-test and Mann–Whitney test were used to compare numerical variables. Analysis of follow-up data were performed by the Kaplan–Meier method, and groups were compared with log-rank test. Univariable analysis were performed for each variable with recurrence and survival. The following variables were included in the univariable analysis: age, sex, body mass index, predicted postoperative first second of forced expiratory volume, PS, history of smoking, comorbidity, previous lung cancer surgery, clinical stage, histology, surgical approach, type of operation, length of stay, adjuvant chemotherapy, postoperative complications. Significant (*P* < 0.05) variables at the univariable analysis, were investigated in multivariable analysis using the stepwise Cox regression. We performed propensity score matching 1 by 1 with caliper width of 0.2 of standard deviation; we included age, sex, clinical stage and previous lung cancer surgery. A stratified cox regression analysis using propensity score matching was performed.

## RESULTS

### Characteristics of patients

Of 3659 patients operated on from April 2007 to December 2019, 253 patients were upstaged from cT1N0 to pT2aN0, because of VPI. The median age was 65 years (IQR: 58–71.5), most patients were male 61.3% (155 of 253). There was no significant difference for age, sex, pack-year smoking, comorbidities, tumour histology, adjuvant chemotherapy between lobectomy and segmentectomy groups. Previous lung cancer surgery (*P* < 0.001), predicted postoperative first second of forced expiratory volume (*P* = 0.009) and clinical stage (*P* = 0.001) especially stage IA1 (*P* = 0.002) and stage IA3 (*P* = 0.001) were significantly different between both arms. Adenocarcinoma was the most prevalent histology (211 patients, 83%) (Table 1). After propensity score matching, patients were grouped *n* = 50 in each team (Table 2).

**Table 1:** Patients and tumours characteristics stratified by type of operation before propensity score matching

	Lobectomy (*n* = 191)	Segmentectomy (*n* = 62)	*P-*Value
Age, median (IQR)	64 (58–71)	67 (59–72)	0.42
Sex, male/female, *n* (%)	119 (62.3)/72 (37.7)	36 (58.1)/26 (41.9)	0.65
ppoFEV1, median (IQR)	69.7 (60–77.2)	80.1 (62.4–93.6)	0.009
PS			0.85
0, *n* (%)	134 (70.2)	44 (71.0)	
1, *n* (%)	53 (27.7)	16 (25.8)	
2, *n* (%)	4 (2.1)	2 (3.2)	
Comorbidity			0.24
No comorditity, *n* (%)	23 (12)	7 (11.3)	
Cardiovascular and pulmonary, *n* (%)	43 (22.5)	18 (29.0)	
Oncological, *n* (%)	19 (9.9)	11 (17.7)	
Others,^a^*n* (%)	105 (54.9)	26 (41.9)	
Previous NSCLC surgery, *n* (%)	2 (1.0)	10 (16.1)	<0.001
Clinical stage			0.001
cT1aN0, *n* (%)	7 (3.7)	9 (14.5)	0.002
cT1bN0, *n* (%)	91 (47.6)	37 (59.7)	0.10
cT1cN0, *n* (%)	93 (48.7)	16 (25.8)	0.001
Histology			0.21
Adenocarcinoma, *n* (%)	155 (81.2)	56 (90.3)	
Squamous, *n* (%)	25 (13.1)	6 (9.7)	
Larger cells carcinoma, *n* (%)	8 (4.2)	0 (0%)	
Others,^b^*n* (%)	3 (1.6)	0 (0%)	
Adjuvant chemotherapy, *n* (%)	37 (19.4)	8 (12.9)	0.33

a Diabetes mellitus, exposure to asbestos, alcoholism, scleroderma or other comorbidities association.

b Sarcomatous carcinoma, lymphoepithelioma-like carcinoma, carcinoid tumour.

IQR: interquartile range; NSCLC: non-small-cell lung cancer; ppoFEV1: predicted postoperative first second of forced expiratory volume; PS: performance status.

**Table 2:** Patient and tumour characteristics stratified by type of operation after propensity score matching

	Lobectomy (*n* = 50)	Segmentectomy (*n* = 50)	SMD
Age, mean (±SD)	64.3 (±10.8)	65 (±10.3)	0.113
Sex male/female, *n* (%)	24 (48.0)/26 (52.0)	24 (48.0)/26 (52.0)	0.055
ppoFEV1, mean (±SD)	65.7 (±16.7)	78.9 (±22.6)	
PS			0.039
0, *n* (%)	35 (70.0)	36 (72.0)	
1, *n* (%)	14 (28.0)	13 (26.0)	
2, *n* (%)	1 (2.0)	2 (2.0)	
Comorbidity			0.201
No comorbidity, *n* (%)	5 (10.0)	7 (14.0)	
Cardiovascular and pulmonary, *n* (%)	12 (24.0)	13 (26.0)	
Oncological, *n* (%)	6 (12.0)	9 (18.0)	
Others,^a^*n* (%)	27 (54.0)	21 (42.0)	
Previous NSCLC surgery, *n* (%)	1 (2.0)	1 (2.0)	0.000
Clinical stage			0.033
cT1aN0, *n* (%)	7 (14.0)	6 (12.0)	
cT1bN0, *n* (%)	31 (62.0)	32 (64.0)	
cT1cN0, *n* (%)	12 (24.0)	12 (24.0)	
Histology			0.356
Adenocarcinoma, *n* (%)	40 (80.0)	45 (90.0)	
Squamous, *n* (%)	7 (14.0)	5 (10.0)	
Larger cells carcinoma, *n* (%)	3 (6.0)	0 (0)	
Adjuvant chemotherapy, *n* (%)	6 (12.0)	7 (14.0)	0.059

a Diabetes mellitus, exposure to asbestos, alcoholism, scleroderma or other comorbidities association.

NSCLC: non-small-cell lung cancer; ppoFEV1: predicted postoperative first second of forced expiratory volume; PS: performance status; SD: standard deviation; SMD: standardized mean difference.

### Perioperative results

Complications occurred in 105 patients (41.5%) over the 90 postoperative days. Thirty-four patients (13.4%) did have a major complication. Ninety-days complications rate was not statistically different between lobectomy and segmentectomy groups. However, length of stay was statistically (*P* = 0.013) shorter in the segmentectomy group, median = 5 days (IQR: 4–6) than in the lobectomy group: median = 6 days (IQR: 4–9).

### Survival

The median follow-up was 41 months (IQR: 24–65.5), it was 38 months (IQR: 25–68) for lobectomy and 43.5 months (IQR: 23–61.75) for segmentectomy. 84 (33.2%) patients had an oncological event (either a recurrence or a second primary lung cancer).

There was no statistical difference in 5-year DFS rate (*P* = 0.37) between lobectomy 70% (95% CI: 60.8–77.5%) and segmentectomy 64.8% (95% CI: 47.2–77.8%) groups.

**Figure 1:**
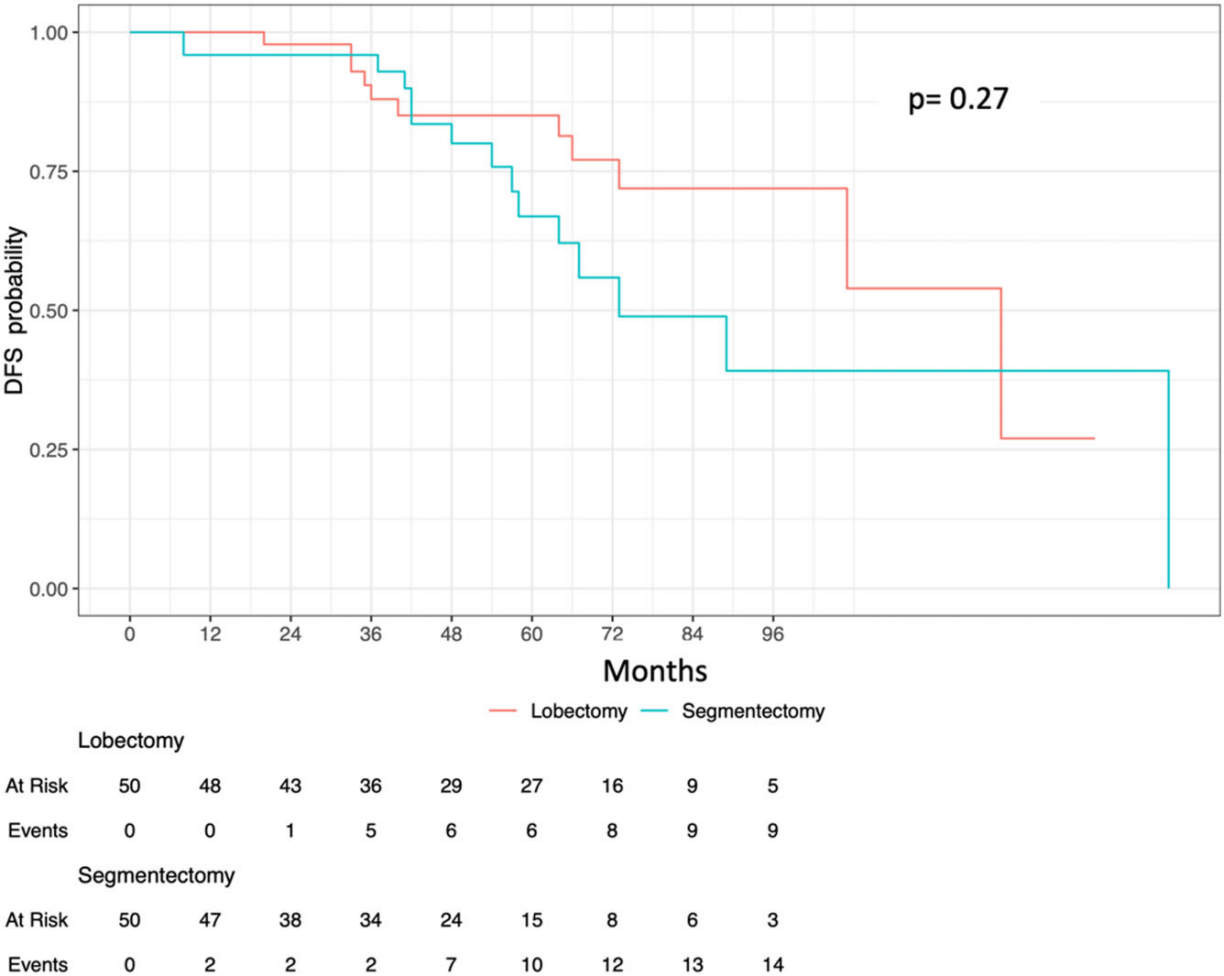
Disease-free survival.

After propensity score matching, the 5-year DFS (*P* = 0.27) rate was similar between lobectomy 85% (74.6–96.9%) and segmentectomy 66.9% (51.3–87.2%; Fig. 1).

Age (*P* = 0.04), PS (*P* < 0.001), previous lung cancer surgery (*P* = 0.05), clinical stage (*P* = 0.04), adenocarcinoma histology (*P* = 0.007), were statistically influencing recurrence in univariable analysis. The type of operation was not influencing recurrence (*P* = 0.74), in univariable analysis even in the matched dataset. After multivariable analysis, only PS [*P* < 0.001, hazard ratio (HR) = 2.41] and age (*P* = 0.031, HR = 1.03) were statistically influencing recurrence.

After stratified Cox regression analysis using propensity score matching, only PS was statistically influencing recurrence (*P* < 0.001, HR = 4.24).

The loco-regional recurrence rate (*P* = 0.84), ipsilateral pleural recurrence rate (*P* = 0.92) and brain recurrence rate (*P* = 0.68) were similar in the 2 arms. However, distant recurrence rate was statistically different between 2 groups (*P* = 0.027) (Table 3).

**Table 3:** Oncological events stratified by type of operation

	Lobectomy (*n* = 191)	Segmentectomy (*n* = 62)	*P*-Value
Recurrences			
Locoregional, *n* (%)	34 (17.8)	10 (16.1)	0.84
Ipsilateral pleural, *n* (%)	13 (6.8)	4 (6.5)	0.92
Distant, *n* (%)	24 (12.5)	15 (24.2)	0.027
Brain, *n* (%)	9 (4.7)	4 (6.4)	0.68
Second NSCLC, *n* (%)	19.9 (9.9)	4 (6.4)	0.40

NSCLC: non-small-cell lung cancer.

**Figure 2:**
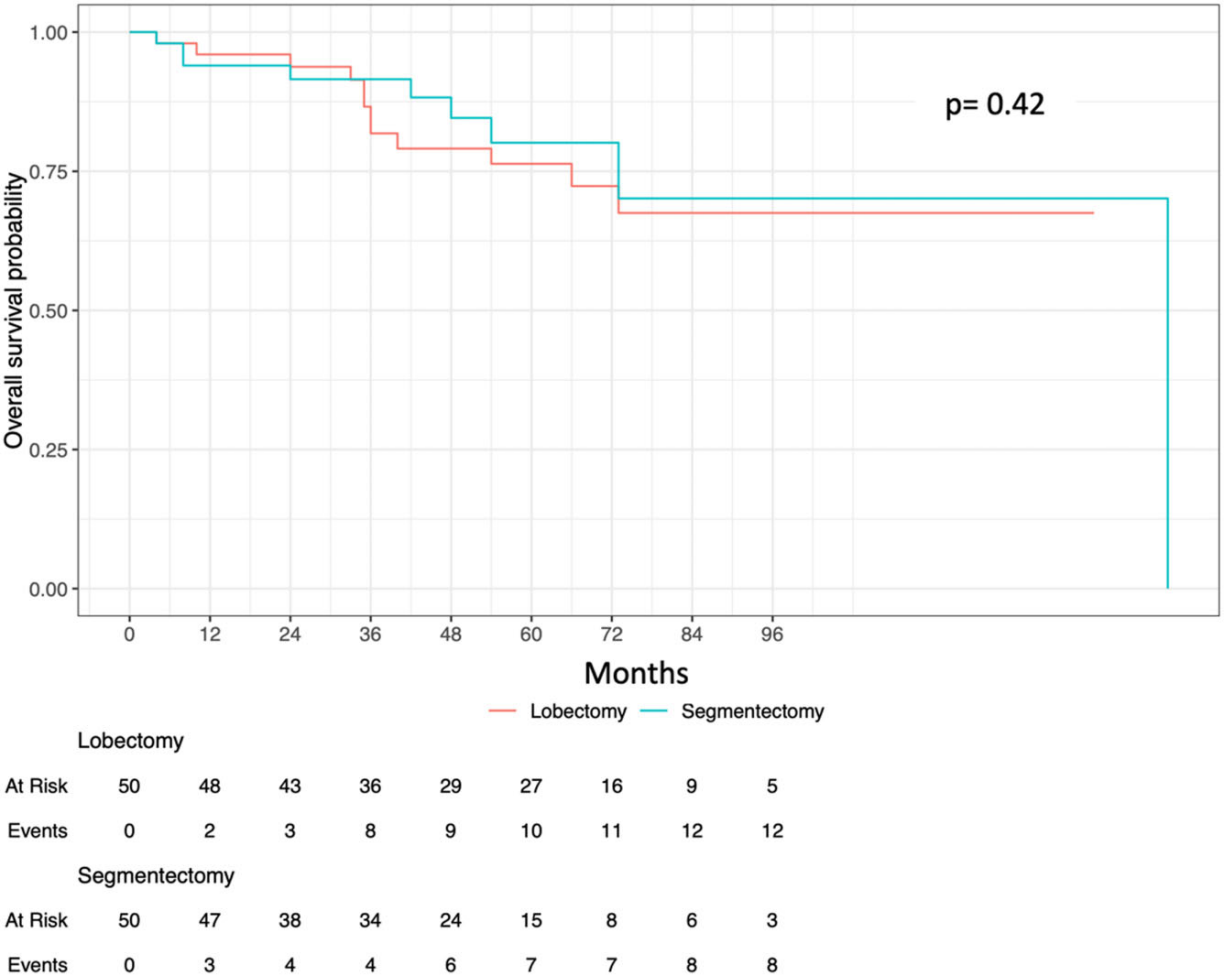
Overall survival.

The 5-year OS rate was similar (*P* = 0.93) between the groups: segmentectomy 75.8% (95% CI: 59.3–86.4%) vs lobectomy 73% (95% CI: 64.4–79.9%). After propensity score matching, the 5-year OS rate (*P* = 0.42) was similar between lobectomy 76.3% (95% CI: 64.4–90.5%) and segmentectomy 80.1% (67.2–95.5%) (Fig. 2).

The following variables were statistically influencing survival in univariate analysis: male gender (*P* = 0.01), PS (*P* < 0.001), length of stay (*P* = 0.001), comorbidities (*P* = 0.013), previous lung cancer surgery (*P* = 0.037) and histology (adenocarcinoma) (*P* = 0.025). The survival was similar (*P* = 0.55) between patients who did have adjuvant chemotherapy (68.9%) and those who did not (79.8%). In multivariable analysis, male gender (*P* = 0.053, HR = 1.83), PS (*P* < 0.001, HR = 3.85) and length of stay (*P* = 0.021, HR = 1.04), were statistically influencing survival.

After stratified cox regression analysis using propensity score matching, only PS was statistically impacting survival (*P* < 0.001, HR = 5.96).

## DISCUSSION

One main limitation in VPI studies is the assessment of VPI itself. In its proposals of the 8th TNM staging, the International Association for Study of Lung Cancer classified the extent of VPI as following: pleura (PL) 0 when invasion is beneath elastic layer, PL1 when invasion is beyond the elastic layer, PL2 when invasion extends to the visceral pleural surface [18]. According to the Japan Lung Cancer Society, for small-sized tumour with VPI, only PL2 should be upstaged to pT2a (stage IB), while PL1 tumours remain T1 (stage IA). In our series, as in most others, PL1 and PL2 were upstaged (pT2a) [12, 19]. Elastic stains are recommended for the diagnosis of VPI, it increases up to 20% the identification of VPI compared to haematoxylin eosin [9, 12, 20], even though many pathologists have been reluctant to use elastic stains [20]. In fact, even with elastic stains, the diagnosis of VPI is not always evident, because of anatomical and pathological variations of visceral pleural caused by inflammation and fibrosis [9].

VPI has been known as an adverse prognostic factor for NSCLC as it correlates with, higher recurrence rate and poor survival [12], even in case of small-sized NSCLC [7, 8].

The question raises when at final pathological examination, VPI upstages from cT1N0 to pT2aN0 NSCLC in patients who did have only segmentectomy; should these patients be reoperated for completion to lobectomy?

They are several series with VPI upstaged pT2a patients who underwent only segmentectomy [1–5, 14, 19, 21], without mentioning any completion to lobectomy; some authors [5, 14] have suggested adjuvant treatment for these patients as an alternative.

We therefore investigated the prognosis of early-stage NSCLC (cT1N0) patients who underwent either lobectomy or segmentectomy with VPI at final pathological examination (upstaged pT2aN0) in standard practice, outside of study protocol over 3 experienced French centres. We found that 5-year DFS and OS rates were similar between the 2 groups; segmentectomy did not increase loco-regional recurrence (including ipsilateral pleural recurrence), second primary lung cancer compared to lobectomy. In multivariable analysis, segmentectomy was not associated with an increased recurrence rate neither with a worse survival. Our results were comparable with those of Kagimoto *et al.* who investigated oncological outcome of segmentectomy for small-sized NSCLC in patients with invasive characteristics, including VPI [21]; Moon *et al.* attested the same when they assessed at completion of resection to lobectomy in patients that underwent sublobar resection with VPI or lymphovascular invasion at final pathological examination [22]. But, in our survey, only the distant recurrence rate was statistically increased in the segmentectomy group. A possible explanation would be the higher number of patients with previous lung cancer surgery in this group without being able to determine whether the distant recurrence is attributed to the first or second resected NSCLC.

In our cohort, adjuvant chemotherapy did not prevent recurrence. It was not either improving survival. Huang relies on adjuvant treatment in case of VPI even for stage IA lung cancer [8]. However, they are some nuances rose by Hattori *et al.* who do not recommend adjuvant chemotherapy based on the VPI, in stage IA patients in case of partial ground glass nodules [14]; and by Chang *et al.* who suggested adjuvant chemotherapy in stage IA patients only with PL2 VPI [23]. The benefit of adjuvant treatment for stage IB, has been a matter of discussion.

Even though OS and DFS rates between segmentectomy and lobectomy were statistically similar in our study, the DFS difference increased from the 5th year in favour of lobectomy. We think that any explanation could be found if we reported some variables that are a part of actual clinical practice context, for instance CTR, adenocarcinoma subtype, margin measurement.

There is a real need of further investigations on the impact of adjuvant treatment, CTR, adenocarcinoma subtype, VPI extent in VPI pT2a upstaged patients with the current clinical settings. Notwithstanding, a conservative attitude upon VPI pT2a upstaged patients after segmentectomy, seems promising.

### Limitations

Several limitations of our study must be underlined, the limited size of the sample and those generally related to its retrospective nature and to its large span time coverage, especially the lack of radiological pattern, surgical margins details and of adenocarcinoma subtypes. Nevertheless, we have not found any argument of extending resection in VPI pT2a upstaged patients who underwent segmentectomy.

## Data Availability

The data underlying this article will be shared on reasonable request to the corresponding author.

## ABBREVIATIONS

DFS: Disease-free survival
HR: Hazard ratio
IQR: Interquartile range
NSCLC: Non-small-cell lung cancer
OS: Overall survival
PL: Pleura
PS: Performance status
TNM: Tumour, node, metastasis
VPI: Visceral pleural invasion
